# The Response of Chromosomally Engineered Durum Wheat-*Thinopyrum ponticum* Recombinant Lines to the Application of Heat and Water-Deficit Stresses: Effects on Physiological, Biochemical and Yield-Related Traits

**DOI:** 10.3390/plants12040704

**Published:** 2023-02-05

**Authors:** Gloria Giovenali, Ljiljana Kuzmanović, Alessandra Capoccioni, Carla Ceoloni

**Affiliations:** Department of Agriculture and Forest Sciences (DAFNE), University of Tuscia, 01100 Viterbo, Italy

**Keywords:** wild wheat relatives, abiotic stresses, stress combination, stress tolerance, stress physiology, proline content

## Abstract

Abiotic stress occurrence and magnitude are alarmingly intensifying worldwide. In the Mediterranean basin, heat waves and precipitation scarcity heavily affect major crops such as durum wheat (DW). In the search for tolerant genotypes, the identification of genes/QTL in wild wheat relatives, naturally adapted to harsh environments, represents a useful strategy. We tested three DW-*Thinopyrum ponticum* recombinant lines (R5+, R112+, R23+), their control sibs lacking any alien introgression, and the heat-tolerant cv. Margherita for their physiological, biochemical and yield response to heat stress (HS) application at anthesis, also in combination with water-deficit stress applied from booting until maturity. Under HS, R5+ and R112+ (23%- and 28%-long 7el_1_L *Th. ponticum* chromosome segment distally inserted on DW 7AL, respectively) showed remarkable stability of the yield-related traits; in turn, R23+ (40%-long 7el_1_L segment), despite a decreased grain yield, exhibited a greater spike fertility index and proline content in spike than its control sib. Under water-deficit + HS, R5+ showed the highest increment in water use efficiency and in flag leaf proline content, accompanied by the lowest yield penalty even vs. Margherita. This research confirms the value of harnessing wild gene pools to enhance DW stress tolerance and represents a starting point for elucidating the mechanisms of *Thinopyrum* spp. contribution to this relevant breeding target.

## 1. Introduction

Global population growth, coupled with reduced availability of arable land and water, are all critical challenges to urgently deal with in order to cope with food security. Projected objectives indicate the need to reach a 60% production increase for staple crops by 2050, and doubled yields for wheat in particular [1]. However, climate changes pose increasing threats to plant vitality and fertility. Under the different climate scenarios of the second half of this century, global cereal production is projected to decrease by 1–11% [2], and, as for wheat, each degree-Celsius of temperature increase is estimated to imply a 6% decline of global yields [3]. Particularly affected by exacerbating weather conditions is the Mediterranean region, where heat waves accompanied by drought phenomena are foreseen to be more and more severe and frequent [4], resulting in the conspicuous loss of crop production and increased yield volatility [5,6]. In the Mediterranean and bordering Middle East countries, a large proportion of the total wheat surface is covered by durum wheat (*Triticum durum* L., 2n = 4x = 28), a species that traditionally provides important social and commercial benefits to the whole area [7,8,9]. In fact, durum wheat (DW) produced in this area accounts for more than half of the global production [2,10,11]. To assure profitable and stable economic returns to farmers and the industry of traditional as well as emerging markets (e.g., [7]), major efforts are needed to counter the projected DW yield losses due to a warmer and drier climate. Such losses are expected to range from −8% to −50% in the Mediterranean area [2]. Releasing varieties with high and stable yields under unfavourable environmental conditions is therefore a primary breeding objective in the Mediterranean regions. However, the required genetic diversity for meaningful interventions is very limited in the extant crop gene pool, thus resulting as insufficient to adequately respond to current and future challenges. On the other hand, wild wheat relatives are still endowed with ample and largely untapped diversity, allowing them to withstand a wide range of harsh environmental conditions [12,13]. A strong diversity reduction emerges from comparison of DW with its closest wild ancestor, *T. dicoccoides* [14,15]. This member of DW primary gene pool, possessing the same AB genomes as the crop species, and so diploid wild relatives sharing similarity with either genome, allow easy access to their adaptive attributes. Hence, they have been rather extensively and successfully harnessed in DW breeding programs addressing yield stability under abiotic stresses [16,17,18].

A further and major novelty potentially able to generate climate-resilient wheat cultivars consists of the utilization of the little-explored genetic diversity present in wild grasses belonging to the wheat tertiary gene pool. To access the defined chromosomal regions and target genes of such more distantly related species, such as those belonging to the *Thinopyrum* genus, pre-breeding approaches referred to as “chromosome engineering” are required [19,20]. Through this strategy, we have recently transferred into bread and durum wheat defined segments of *Thinopyrum* chromosomes, specifically of decaploid *Thinopyrum ponticum* (Popd.) Barkworth and D.R. Dewey (2n = 10x = 70) and, in a “nested” fashion within the above ones, of diploid *Thinopyrum elongatum* (Host) D.R. Dewey (2n = 2x = 14). The target traits for introgression were primarily resistances to major wheat diseases, namely leaf rust, stem rust and Fusarium head blight [19,20,21,22]. However, when field trialled across contrasting environments (including abiotic stress-prone) and water-regimes, such DW-*Thinopyrum* spp. recombinant lines revealed improved yield-contributing traits and higher yield stabilities than the control lines lacking the alien segment(s) [23,24]. This evidence was suggestive of a potential involvement of these *Thinopyrum* spp. segments in abiotic stress tolerance. However, while better known for their ability to withstand salinity stress [25], this group of species has been less explored and characterized for heat and drought tolerance [26,27]. Certainly, little knowledge is available of specific stress tolerance/adaptation mechanisms and of the possible association of the tolerant phenotype(s) with the presence of particular alien segments [28,29].

Plants, as sessile organisms, cannot escape from unfavourable environments; therefore, the ability to activate effective adaptive mechanisms against abiotic stress holds great importance in view of yield preservation. Both high temperature peaks and heat waves occurring during the reproductive phase, particularly at flowering, are detrimental to wheat yield formation, with excess temperatures above the optimal 21 °C leading to floret abortion, pollen sterility and finally decreased grain number and yield [30,31]. Similarly, water-deficit occurrence around anthesis affects many key traits related to spike and plant fertility, such as fertile florets per spikelet and grain number per spike and per plant [32,33]. Likewise, drought at the grain filling stage leads to a reduction of grain weight, grain yield and harvest index [34]. On the other hand, under field conditions, heat and drought phenomena often occur simultaneously and usually aggravate the negative impacts of single stresses on crop yield; furthermore, the plant reaction to multiple stress combinations is hardly predictable, and cannot be directly extrapolated from the response to individual stresses [35,36,37].

The drop in spike fertility and other yield traits under hot and dry conditions is strongly related to physiological and biochemical perturbations occurring in stressed plants. As in practically all green plants, photosynthetic efficiency is highly reduced in wheat subjected to high temperatures and water limitations, due to accelerated leaf senescence, which restricts green-photosynthetic areas, as well as to disruption of chloroplasts’ structure and function, implying reduction of pigments such as chlorophyll and carotenoids, inactivation of key enzymes and membrane injury (e.g., [38] and references therein). Hence, chlorophyll fluorescence analysis, which enables the assessment of several parameters related to photosynthetic efficiency, is a commonly used tool to monitor the physiological status of plants under abiotic stress conditions [39,40,41]. 

Stomata, determining the rate of CO_2_ uptake in leaves and, consequently, the amount available at the Rubisco site through their opening/closing mechanism, are key players in photosynthetic efficiency and plant transpiration [42]. Genotypic variation and a positive relation with grain yield have been reported for stomatal conductance in wheat under well-watered conditions [43]. Under prolonged heat occurrences, stomatal conductance is usually enhanced to promote evaporative cooling, thus reducing the inner temperature [44,45]. However, there is also evidence of stomata closure under heat exposure, suggesting a fine and sensitive regulation of this physiological mechanism, subject to intra- and inter-specific variations [46]. In water-limited conditions, stomata closure helps reduce water loss and maintain cell turgor, but leads to decreased CO_2_ influx and carbon fixation hindrance in the Calvin cycle [47,48]. As a result, chloroplasts become exposed to excessive excited energy and enhance the production of toxic reactive oxygen species (ROS), which, in turn, cause oxidative damage [49].

In addition to oxidative damage, both drought and heat phenomena trigger a secondary osmotic stress in plants, resulting in the loss of cell turgor. To overcome this, a wide range of molecules known as compatible solutes, including sucrose, trehalose, glycine, betaine and proline, can accumulate at high concentrations in tissues, thus protecting cells against osmotic imbalance. Among them, proline (Pro) is a very versatile compound, whose beneficial effects against different abiotic stresses, not only as an osmolyte, but also as a stabilizer of sub-cellular structures, buffer of cellular redox potential, ROS scavenger, signal molecule and protein folding chaperone, are widely demonstrated [50,51]. An increment of Pro content has been consistently reported in the leaves of heat- or drought-stressed wheat plants, and the activation of this defence mechanism was strongly correlated with a higher stress tolerance (e.g., [52,53]). However, less is known about Pro accumulation in sink organs, such as spikes, directly related to yield, and about its role in plants subjected to heat stress and water-deficit simultaneously.

In view of exploiting novel and exotic germplasms for improving the abiotic stress resilience of wheat crops, it is particularly relevant to study how wild gene introgressions impact the critical defence parameters and ultimately the yield of the recipient crop species. To this aim, and to validate the preliminary evidence from field trials [23,24], in the present study we have evaluated, under controlled conditions, the performance of DW-*Th. ponticum* near-isogenic recombinant lines (NIRLs), containing differently sized fractions of the alien 7el_1_L chromosome arm, when subjected to heat stress alone and in combination with water-deficit stress. Physiological and biochemical parameters of leaf and spike tissues, as well as several plant and spike yield-contributing traits, were assessed. As a result, thanks to the type of the experimental materials employed, the research allowed us to associate the observed phenotypic changes following the single and combined stress application to specific 7el_1_L regions, and hence to highlight the best performing recombinant lines as candidates for stress-tolerant DW breeding. 

## 2. Materials and Methods

### 2.1. Plant Materials

**Figure 1:**
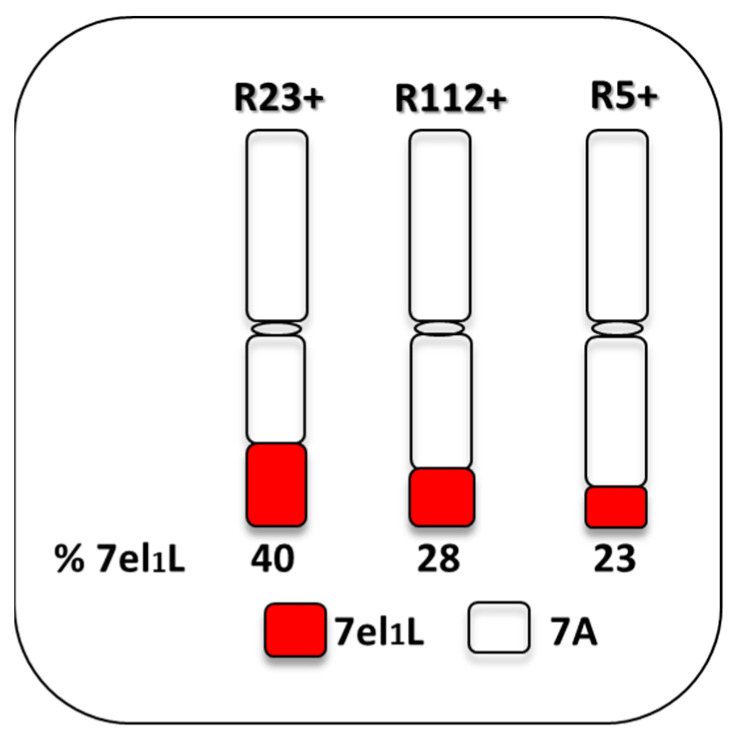
Durum wheat-*Thinopyrum ponticum* recombinant lines tested under heat and water-deficit conditions. 7el_1_L: chromosome segment derived from *Th. ponticum* and transferred via a chromosome engineering strategy; 7A: durum wheat recipient chromosome.

Three DW-*Th. ponticum* NIRLs were employed in the present study, named R5-2-10, R112-4 and R23-1 [54], and are here referred to as R5+, R112+ and R23+, respectively. They were developed in the background of the Italian DW cv. Simeto by repeated backcrossing (BC_5_ for R5+ and R112+ and BC_4_ for R23+) followed by several self-generations. The NIRLs have different portions of the *Th. ponticum* 7el_1_L chromosome arm on the distal end of the recipient DW 7AL arm (Figure 1). The symbol “+” indicates homozygous carriers of the respective 7el_1_L segment, while homozygous sib lines, non-carriers of the given 7el_1_L segment, are accompanied by the “−” symbol. An additional DW genotype used was the ICARDA cv. Margherita, introduced in the analysis because it expressed a remarkable heat tolerance ability in a range of stressful environments, including the Senegal River Basin, characterized by severe temperature extremes during the flowering stage [55].

### 2.2. Plant Growing Conditions and Stress Treatments

Two separate experiments were carried out to investigate plant behaviour towards heat stress (HS) alone or combined with water-deficit and heat stress (WH). For both experiments, sterilized seeds were placed to germinate on moistened filter paper (22–24 °C), and then transferred in pots containing soil, sand and perlite in a 6:1:1 ratio for vernalization in a cold cabinet at a 8°/6 °C (day/night) temperature and 12 h photoperiod for 15 days. Afterwards, the plants were moved into a walk-in controlled environment chamber, where they were given varying temperature and light regimes, depending on the phenological state and the treatment conditions. The plants were uniformly fertilized and watered, except for those subjected to the water retention treatment (see below).

**Figure 2:**
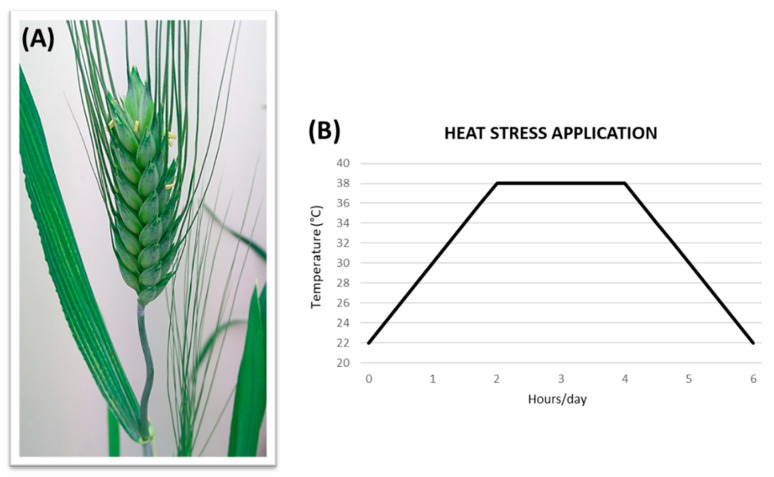
Heat stress application: (**A**) Phenological phase of the main tiller at the time of stress application, corresponding to anthesis (Zadoks scale 61–65). (**B**) Procedure followed for heat treatment, performed for three consecutive days in the heat stress experiment, and for three or seven consecutive days in the combined heat + water-deficit stress experiments.

**Figure 3:**
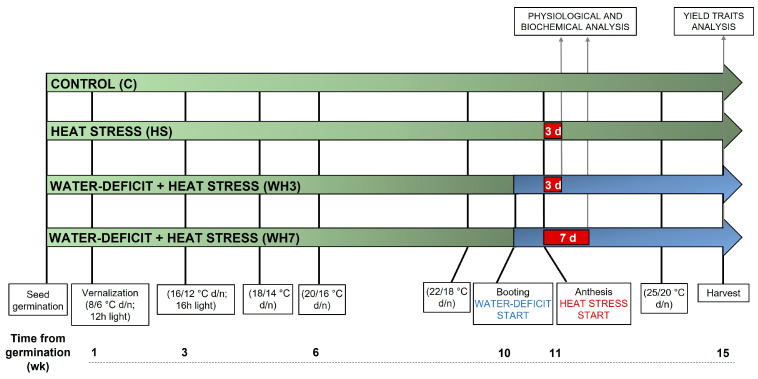
Timeline and features of experimental plant growth conditions, stress treatments application and data recording. Approximate duration (in weeks, wk) of main growth intervals and of the total cycle since seed germination is indicated. d = days.

In the HS experiment, to simulate field conditions, plants whose main culm had reached anthesis (Zadoks scale 61–65, [56]; Figure 2A) were subjected to a gradual increase in temperature in a dedicated chamber. The treatment consisted of a progressive increase in temperature over a 2 h interval, from 22 °C up to 38 °C (+2 °C every 15 min), followed by 2 h at 38 °C and by 2 h of a gradual decrement from 38 °C to 22 °C (−2 °C every 15 min), totalling 6 h/day of HS applied over 3 consecutive days (Figure 2B). After the HS application, the plants were brought back to the original chamber and kept there until harvest. In the same chamber, the control (non-stressed) plants were maintained at 22 °C/18 °C during anthesis, and the whole set of materials were subsequently grown as illustrated in Figure 3.

In the combined stress (WH) experiment, water withholding started at the booting stage (about 10 weeks from germination) and ended at maturity, mimicking a prolonged stress condition. At anthesis, HS was applied as described above on two subsets of plants for either 3 (WH3) or 7 (WH7) consecutive days (3d, 7d; Figure 3). For the water-deficit application, the pots were initially watered to maximum capacity, left to drain overnight and then the soil surface was covered with an aluminium foil to avoid soil evaporation. The pot weight upon drainage completion was taken as 100% Soil Water Content (SWC). The pots were weighed every 2 days to maintain the stressed plants at 30% SWC and the control plants at 70% SWC. Fifteen–twenty plants per genotype and experimental condition (control and stress) were grown and used for data recording, totalling about 300 plants in each of the two experiments.

### 2.3. Physiological and Biochemical Measurements

#### 2.3.1. Physiological Parameters

Measurements of the physiological parameters were taken on 10 stressed and control plants after the 3rd day of stress application in the HS, and after the 3rd and 7th day in the combined WH stress experiments. Chlorophyll content (SPAD) was measured on 3 positions along the adaxial surface of flag leaf (FL) using a hand-held chlorophyll meter (SPAD- 502 Plus; Konica-Minolta, Japan). Stomatal conductance (SC, mmol/[m^2^s]) and leaf temperature (TL, °C) were measured in the middle of the FL adaxial surface using a SC-1 leaf porometer (METER Group, Inc. USA). To evaluate the photochemical response of plants, the OJIP curve (Fluorescence Transient) was recorded using a portable fluorometer (PAR-FluorPen FP110; PSI- Photon Systems Instruments, Drásov, Czech Republic) on the FL adaxial surface after a dark adaptation for 30 min with detachable clips. The OJIP curve provided parameters of the status and function of photo-system II (PSII) reaction centres, such as Fv/Fm (Max photochemical efficiency), Fv/F0 (Potential photochemical efficiency) and Performance Index (PI). The latter expresses the energetic bifurcations of PSII [39,40,41], being a combined measure of the quantity of the photosynthetic reaction centres (RCs), maximum energy flow reaching the RCs and electron transport at the beginning of illumination.

Relative water content (RWC) was assessed on 5 fully expanded penultimate leaves (FL-1) of the main culm per genotype and treatment and calculated as RWC = (FW-DW)/(TW-DW), where FW, DW and TW stand for fresh, dry and turgid leaf weight, respectively. Namely, a 5 cm-long segment from the middle portion of each FL-1 was excised, its FW recorded and then put at 4 °C overnight in tubes with cut ends immersed in distilled water for the next day’s TW measurement. Finally, turgid leaf segments were oven-dried for two days at 65 °C to assess DW.

In the WH experiments, water use (WU) and water use efficiency (WUE) were also evaluated on 5 plants per genotype and treatment. First, the WU of the control and stressed plants was obtained by quantifying the total amount of water (g) given to each pot from the beginning of drought stress application (booting stage) until plant maturity, and was normalized per plant dry biomass unit. The amount of water added to each pot was calculated on the basis of differences in pot weight, so as to maintain the 30% or 70% SWC for stressed or control plants, respectively, as described above. Second, the water use efficiency (WUE) was calculated at harvest as the ratio between plant grain yield and WU [57,58]. 

#### 2.3.2. Proline Quantification in Leaves and Spikes

Flag leaf (FL) and spike (awns excluded) sampling was performed immediately after stress application; tissues were collected in tubes, suspended in liquid nitrogen and stored at −80 °C until use. Proline (Pro) content was measured in FL (Pro-FL) and spikes (Pro-SP) of stressed and control plants following a standardized ninhydrin-based colorimetric assay [59]. The absorbance of 200 μL of the obtained Pro extract was read on a 96-well plate by a microplate reader at 520 nm, using toluene as a reference. Pro concentration of samples was determined in technical duplicates using a standard concentration curve. Pro content corresponded to µmol/g FW. At least 3 plants/genotypes/treatment conditions were used for Pro quantification.

### 2.4. Evaluation of Yield-Related Parameters

Plant height (H, cm) and above-ground plant dry biomass (BM, g) were measured at harvest. For the main culm, i.e., the one that was subjected to HS exactly at anthesis in both experiments, grain number per spike (GNS1), grain number per spikelet (GNSL1), spike fertility index (SFI1), thousand grain weight (TGW1, g) and grain yield per spike (GYS1, g) were measured. The same parameters were also assessed at the whole plant level (GNS, GNSL, SFI and GYS). The number of tillers per plant (TNP) and of productive tillers per plant (PTP), grain number per plant (GNP), thousand grain weight (TGW), grain yield per plant (GYP) and harvest index (HI) were also recorded on harvested plants. SFI, a spike fertility parameter positively correlated with fruiting efficiency, was calculated as the ratio between GNS and chaff weight [23], and HI as the ratio between GYP and BM. The measurements of all the yield-related parameters were derived from at least 10 plants for each genotype/treatment combination.

### 2.5. Stress Indices

To better evaluate and identify stress-tolerant/susceptible genotypes, the following stress indices were calculated by applying the formulas reported in Table 1 (as from [60]): tolerance index (TOL), mean productivity (MP), geometric mean productivity (GMP), stress tolerance index (STI), harmonic mean (HM), stress susceptibility index (SSI), yield index (YI), yield stability index (YSI) and relative stress index (RSI). All the indices are yield-based and commonly used to estimate the tolerance or susceptibility to abiotic stresses (e.g., [60,61,62]). The Plant Abiotic Stress Index Calculator (iPASTIC) software [60] was used to calculate all indices, by setting the input model with mean data derived from GYP, obtained in both control (Yp) and stress (Ys) conditions. Through the estimation of the average of ranks (AR), based on all indices’ values, iPASTIC also allowed for a comprehensive and reliable ranking of genotypes (the lower the AR value, the more stress tolerant the genotype).

### 2.6. Statistical Analysis

One-way analysis of variance (ANOVA) was performed to estimate the differences ascribable to the genotype (G) effect, while two-factor ANOVA was applied to analyse the effect of the G × treatment (T) interaction. Whenever a significant F value was obtained for single factors or their interaction, a Tukey HSD test was performed at *p* < 0.05 level. All statistical analyses were performed by JASP software (JASP Team, 2022; JASP Version 0.16.1). For all the measured parameters, two statistical comparisons (ANOVA) were performed: in the first one, the datasets of the individual NIRLs (+) and of the corresponding non-carrier sib lines (−) were compared, to associate possible differences in stress behaviour with the presence of a specific alien segment; in the second one, the three NIRLs’ datasets were compared to that of cv. Margherita, to evaluate their performance with respect to a known heat tolerant and productive cultivar.

Pearson’s correlation analysis was performed for a subset of yield-related and physiological traits. Each pair of variables was correlated by calculating Pearson’s correlation coefficients (r value), and four different conditions were separately considered: control, HS, WH3 and WH7. Four principal component analyses (PCA), one for each condition, were also made for NIRLs + and Margherita genotypes in the R Environment (R Project for Statistical Computing 4.2.1), using the missMDA package.

## 3. Results

### 3.1. Heat Stress (HS) Application 

#### 3.1.1. Physiological and Biochemical Response to HS

Heat treatment (T) and genotype differences (G) had a significant individual impact on most of the physiological traits and on proline (Pro) content (Table 2). HS decreased most of the parameters, notably chlorophyll content (SPAD) and stomatal conductance (SC), as shown by both of the statistical comparisons performed (Table 2). The presence/absence of alien segments (G) was significant for SPAD, SC and Pro-SP (Table S1, Tukey test). In particular, a positive difference was detected for SC in R5+ vs. R5- (+33%) and for Pro-SP in R23+ vs. R23- (+55%). In comparison with cv. Margherita, Pro-SP of the R23+ and R112+ also proved to be noticeably superior (+82% and +44%, respectively; Table S2), while the SC of all three recombinants showed lower values (−15% to −36%). 

**Figure 4:**
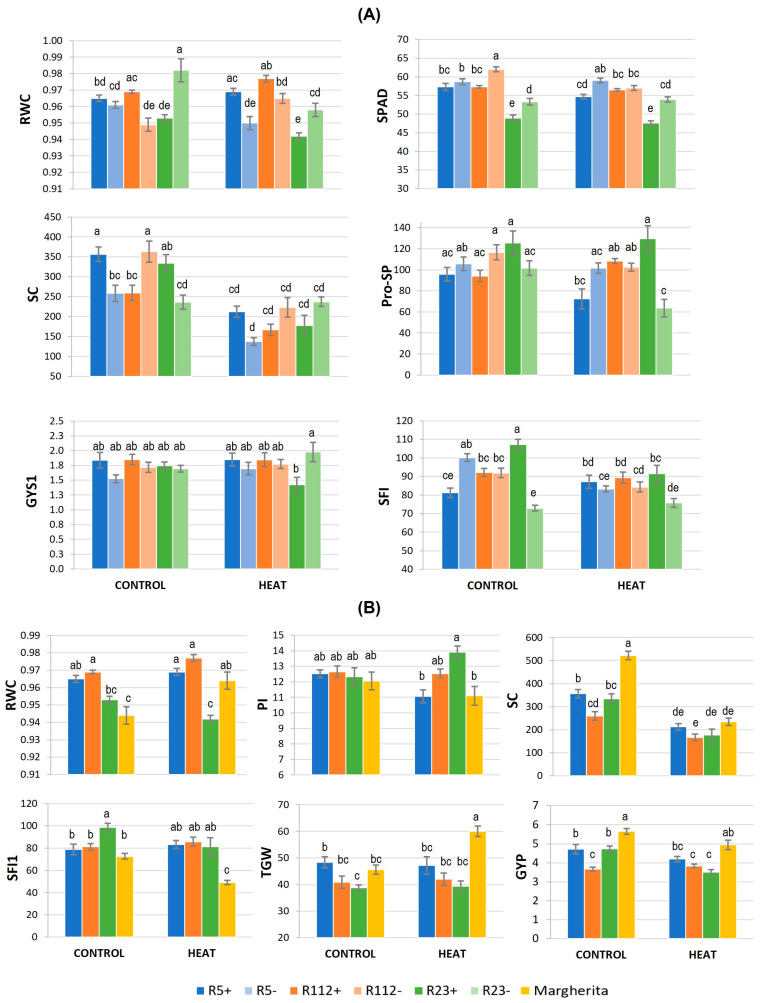
Significant genotype × treatment effects on physiological and yield-related parameters under 3-day heat stress: (**A**) comparison between alien segment carrier (+) and non-carrier (−) NIRLs, and (**B**) comparison between NIRLs+ and cv. Margherita. The error bars represent standard errors of the means, while the letters above histograms correspond to the ranking of Tukey HSD test at *p* < 0.05 significance level. For traits acronyms, see the Materials and Methods section.

The results of the Tukey test for significant G×T interactions (Figure 4 and Tables S3 and S4) showed, as a whole, R5+ and R112+ to have the most tolerant response to HS among NIRLs. This was particularly sustained by their better turgor maintenance vs. the control (“−“) NIRLs (RWC, Figure 4A), comparable to that of cv. Margherita (Figure 4B). Higher leaf hydration under HS of the two recombinants, ascribable to their 7el_1_L segments’ presence, was accompanied by good performance of their photosynthesis-related traits. For example, although the chlorophyll content (SPAD) in R5+ was significantly lower than in R5- under HS (−7%), this did not seem to affect its photosynthetic efficiency, as the chlorophyll fluorescence traits Fv/Fm and PI did not appear to be altered by HS (Table S3). For all physiological traits, R5+ and R112+ showed similar values to those of cv. Margherita, which confirms their positive response to the heat stress (Table S4).

In contrast, the presence of the longest 7el_1_L segment, as in R23+, had a negative impact on both RWC and SPAD, as shown by the observed significant decrease under HS (−2% and −13%, respectively, Table S3). However, a comparison of R23+ values under the HS and control (unstressed) conditions highlighted the ability of this recombinant line to maintain the leaf hydration level of the control samples under HS (RWC, Figure 4A). The same was observed for Pro-SP of R23+, not altered under HS vs. control. Spike Pro content of R23+ resulted as 103% higher under the stress vs. R23- (Figure 4A), which, instead, had a significant 40% decrease in HS vs. control conditions. Other alien segments did not confer noticeable advantages for Pro accumulation in spike or flag leaf, and, in comparison with cv. Margherita, the three recombinants showed similar values for both Pro-FL and Pro-SP (Table S4). 

In the absence of HS, stomatal conductance (SC) was significantly increased by the presence of alien segments in R5+ and R23+ recombinants compared to their negative controls (+38% and +42%, respectively), while the opposite trend was observed in the case of R112+ (−28%) (Table S3 and Figure 4A). In comparison with cv. Margherita, all three recombinants showed 32–50% lower SC in the control condition (Table S4 and Figure 4B), suggesting their constitutively lower leaf gas exchange. However, HS caused a significant reduction in all genotypes (about −40% in NIRLs+ and −50% in Margherita), leading to comparable SC values between them, indicating similar reactions to the stress through this mechanism (Tables S3 and S4 and Figure 4).

#### 3.1.2. Main Fertility and Yield-Related Traits Involved in HS Response

Most fertility traits of the main culm, the one principally targeted by HS at anthesis, were affected by the alien segment presence (G, Table 2A). Variations due to the treatment (T) were only observed in the comparison between NIRLs+ and cv. Margherita (Table 2B). However, G × T interaction was significant for several traits of the main culm, highlighting differences among genotypes in both of the statistical comparisons performed (Table 2A,B). Grain yield of the main culm (GYS1) appeared to be similar across all genotypes in both stress and control conditions (Tables S3 and S4), except for R23+, which had 28% lower GYS1 vs. R23- under stress, the negative effect being thus ascribable to the presence of the 40%-long 7el_1_L segment (Figure 4A). R23+ exhibited significantly higher values for grain number-related traits (GNS1, GNSL1 and SFI1) vs. R23- under control conditions (Table S3). In comparison with cv. Margherita, all the recombinants displayed a higher fertility of the main culm under HS, as proven by their average 70% higher SFI1 (Table S4 and Figure 4B) and, as for R112+, 36% and 26% higher GNSL1 and GNS1, respectively (Table S4). Spikelet fertility (GNSL1) of all recombinants under HS remained similar to that of the control condition (Tables S3 and S4), suggesting a stabilizing role of alien segment presence under stress for this trait.

Similarly to what observed for the main culm, also at the whole plant level R23+ had, under control conditions, a better performance than R23- for SFI (+47%), GNP (+53%) and GYP (+23%), evidently due to its alien segment (Table S3). Under HS, R23+ maintained a 20% higher SFI than R23- (Figure 4A), though not the GYP advantage (Table S3). The presence of the other alien segments did not show significant effects on whole plant yield traits. With respect to control conditions, the heat stressed Margherita plants significantly reduced many traits related to grain number (GNS, GNSL, GNP) and spike fertility (SFI), and were shown to be, on average, 86% inferior to all three recombinants for the latter trait (Table S4). Conversely, under HS, Margherita displayed a significant 31% higher grain weight vs. control conditions (TGW, Figure 4B), which likely enabled its high GYP, despite a conspicuous decrease of GNP (−35%, Table S4; Figure 4B). In the same NIRLs+ vs. Margherita comparison, apart from absolute values (depending on background genotypes), both R5+ and, even more, R112+ revealed a remarkable stability of plant yield traits under HS vs. control conditions, mainly due to the maintenance/increase of grain number and fertility parameters both at the spike and plant level (Table S4).

### 3.2. Combined Water-Deficit and Heat Stress Application

#### 3.2.1. Physiological and Biochemical Response to Combined Stress

The combination of water-deficit (W) and heat (HS) stress (= WH) treatments (T) had, as expected, a significant impact on more traits (Table 3 and Table 4) than those affected by HS alone (Table 2). For both experiments, in which HS was imposed for 3 (WH3) or 7 (WH7) days at anthesis, ANOVA analyses showed the effect of genotype alone (G) to be significant for about half of the physiological traits measured (Table 3 and Table 4). Among the genotype-dependent outcomes, the presence of 7el_1_L chromosome segments negatively affected SPAD of NIRLs R112+ (−11% and −13% in WH3 and WH7, respectively) and R5+ (−9% in WH7) compared to their R112- and R5- control lines (Tables S5 and S9). With respect to cv. Margherita, all three NIRLs+ showed significantly lower water use (WU) (Tables S6 and S10), suggesting a higher water uptake and transpiration of the control cultivar. This was supported by the significantly higher SC of Margherita vs. all NIRLs+ in WH3 (average 23%) and vs. R112+ and R23+ in WH7 (+15%) (Tables S6 and S10). As for traits related to photosynthetic efficiency, i.e., Fv/Fm, Fv/F_0_ and PI, the R112+ recombinant outperformed Margherita in WH3 (Table S6), whereas in WH7, R112+ had 36% lower Pro-FL (Table S10).

**Figure 5:**
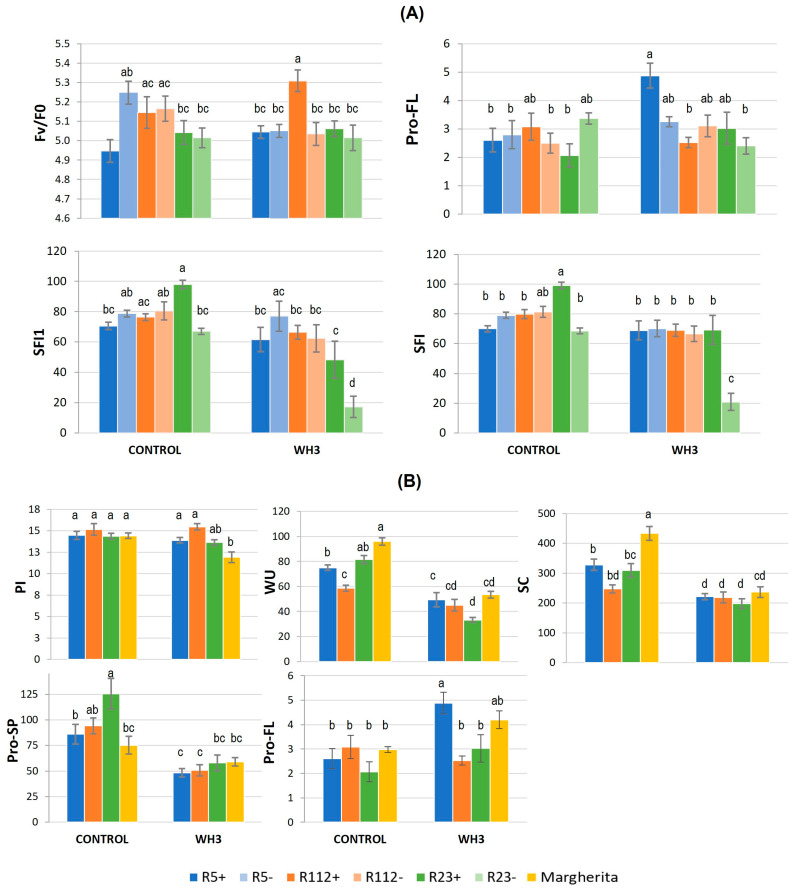
Significant genotype × treatment effects on physiological and yield-related parameters under combined water-deficit and 3-day heat stress (WH3): (**A**) comparison between alien segment carrier (+) and non-carrier (−) NIRLs, and (**B**) comparison between NIRLs+ and cv. Margherita. The error bars represent standard errors of the means, while the letters above the histograms correspond to the ranking of Tukey HSD test at *p* < 0.05 significance level. For trait acronyms, see the Materials and Methods section.

When the G × T interactions were considered, significant differences were observed concerning photosynthesis- and water management-related traits (Table 3 and Table 4). However, only a limited number of significant effects turned out to be conferred by the alien segments (Tables S7 and S11). Under WH3 stress conditions, the 7el_1_L chromosome segment conferred a slight, yet significantly higher PSII potential photochemical efficiency to R112+ compared with R112- (+5% Fv/F0, Figure 5A). Moreover, R112+, as well as R5+, were also better performing than Margherita for PI (+30% and +17%, respectively; Figure 5B), which indicates their greater ability to maintain photosynthetic activity under stress. Comparing WH3 vs. the control conditions, R5+, R23+ and Margherita decreased their WU and SC values more than R112+ (Figure 5B), suggesting the maintenance of water status and water economy to be key mechanisms in their adaptation to the combined stress.

Proline accumulation under combined stress was not as much of a differential between the genotypes as under HS application alone. None of the three alien segments significantly altered the Pro concentration in the WH3 or WH7 experiments (Pro-FL and Pro-SP, Tables S7 and S11). In the WH3 experiment only, R23+ was shown to have a constitutively higher Pro amount in the spike (Pro-SP) than cv. Margherita (+48%) and R5+ (+58%) (Table S6), but the potential advantage was apparently lost under the stress (Table S8 and Figure 5B). As for Pro in the flag leaf (Pro-FL), R5+ nearly doubled its amount under stress vs. control conditions (Table S8 and Figure 5B). A similar behaviour was exhibited by cv. Margherita, whereas R23+ only had a mild increase and R112+ a non-significant reduction.

**Figure 6:**
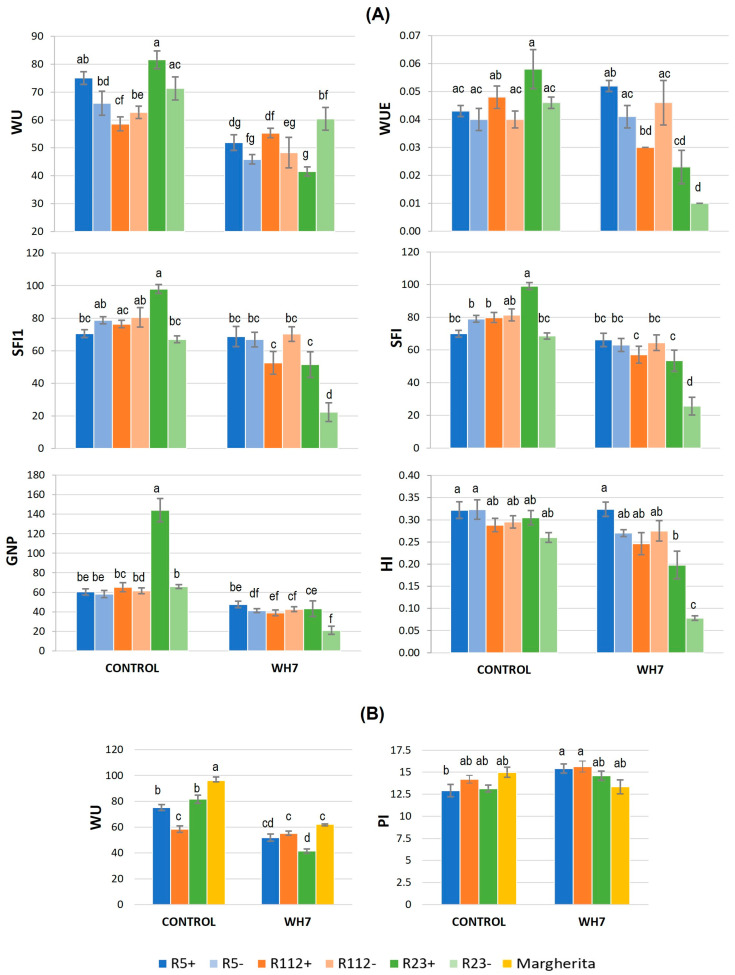
Significant genotype × treatment effects on physiological and yield-related parameters under combined water-deficit and 7-day heat stress (WH7): (**A**) comparison between alien segment carrier (+) and non-carrier (−) NIRLs, and (**B**) comparison between NIRLs+ and cv. Margherita. The error bars represent standard errors of the means, while the letters above the histograms correspond to the ranking of Tukey HSD test at *p* < 0.05 significance level. For trait acronyms, see the Materials and Methods section.

Under more intense heat stress, as in the WH7 experiment, the traits associated with plant water management were shown to be even more important than in WH3 for stress tolerance. Lower values under stress vs. control conditions were in general observed in all NIRLs+ for SC (Table S11). As in the WH3 experiment, R23+ was confirmed to be the best water-saving genotype under stronger stress conditions due to the presence of its 7el1L segment (−50% WU in R23+ and −15% in R23- vs. their respective control conditions, Table S11 and Figure 6A). A significantly decreased WU under stress vs. control conditions was also exhibited by R5+ and cv. Margherita, indicating the good adaptive ability of the three genotypes to a longer exposure to stress, as opposed to R112+, which maintained an unchanged water request in WH7 (Figure 6B and Table S12). As a whole, the comparison with cv. Margherita showed the three NIRLs+ to be similar to the heat tolerant control genotype for most physiological traits under WH7 stress conditions (Table S12). Interestingly, however, R5+, besides the better WU, also showed an increased PI value under stress with respect to the control conditions (Figure 6B and Table S12), which highlights its putative capacity to increase its photosynthetic efficiency under stress.

#### 3.2.2. Influence of Combined Stress on Spike Fertility and Plant Production

Essentially all spike fertility and plant production traits were strongly affected by the stress combination in the WH3 and WH7 experiments, as shown from all statistical analyses performed (T, Table 3 and Table 4). However, seven days of heat treatment (WH7) were more impactful on all genotypes and reduced the differences among them. The significance of the genotype effect alone (G) was also observed for all traits in NIRLs+ vs. NIRLs- analysis (Table 3A and Table 4A), and, for most traits, in NIRLs+ vs. Margherita comparisons (Table 3B and Table 4B). Specifically, in both WH3 and WH7, the effect of the presence of the alien segment was almost exclusively observed for the R23+ recombinant vs. its control line R23-. The effect was remarkably positive for fertility traits of the main culm (average +36% for GNS1 and GNSL1, +71% for SFI1) and of the whole plant (+107% GNP), but also for the tiller number (average +61% TNP and PTP) and grain yield (average +68% GYP) (Tables S5 and S9). In WH3 only, the 40%-long segment of R23+ also increased GNS, GNSL (33–37%) and SFI (76%) at the whole plant level (Table S5), while in WH7, this was the case for HI (+47%) (Table S9). These results indicated that the alien segment present in R23+ constitutively enhances the grain and tiller number, likely directing the allocation of nutrients into grains rather than to biomass. By contrast, the grain weight (TGW) significantly decreased in R23+ vs. R23- in both experiments (−27%; Table S5), while under prolonged HS stress, the TGW of R5+ was 9% higher vs. R5- (Table S9). 

In comparison with Margherita, the Tukey test for the G factor revealed NIRL+ to be not significantly different for the spike grain number and fertility, while differences were more evident for some plant traits (Tables S6 and S10). The results indicated that, with the increase of heat stress intensity, as in WH7, R5+ resulted as more tolerant than R112+, possibly due to the positive impact of its 23%-long 7el_1_ segment on the grain weight under stress (TGW) and to the lower water economy of R112+, as described above (§ 3.2.1). On the other hand, although R23+ had significantly lower TGW, TGW1 and GYS1 than Margherita under both WH3 and WH7, this recombinant appeared to be similar to the heat tolerant cultivar for the plant yield components TNP, PTP, BM and the final yield (GYP) (Tables S6 and S10). This was likely due to R23+ having significantly higher SFI (+23 in WH7, +29% in WH3) and also GNP (+20%) in WH7, confirming that the higher spike fertility of R23+ determines its productivity under combined stress conditions. 

For the majority of traits, ANOVA indicated a significant G × T interaction, especially in the comparison between the NIRLs (Table 3A and Table 4A). Overall, while in the WH3 condition R23+ was shown to be the most affected by the stress (comparisons between control and stressed values) for a number of traits (−30% to −70% for GNS1, GNSL1, SFI1, GYS1, GNS, GNSL, SFI, TNP, PTP, GNP, BM, GYP; Table S7), in the WH7 experiment, this was also true for R112+. On the other hand, R5+ plants proved to be rather stable between the control and stress conditions, only displaying the negative stress effect on the spike grain number in WH7 (GNS, GNSL, Table S11). The only alien segment associated with a significant increase under the combined stress was that of the R23+ recombinant. In both stress experiments, extraordinarily 1–2.3-fold higher values of SFI1 and SFI were displayed by R23+ in comparison with R23- (Tables S7 and S11, Figure 5A and Figure 6A). In WH7 only, the same was observed for the total grain number/plant (GNP), 1.2-fold higher in R23+ than in R23- (Table S11 and Figure 6A). SFI1, SFI and GNP were the only traits that under both stress and control conditions confirmed the positive association between spike fertility (and consequently total grain number/plant) and the presence of the 40%-long Th. ponticum chromosome segment. Considering the three NIRLs+ vs. Margherita, their response to stress was highly similar to that of the cultivar (Tables S8 and S12), with no depressing effect on the yield traits vs. control values (particularly in WH3). In WH3, only R23+ showed a remarkably lower grain number of the main culm (−37% GNS1 and −40% GNSL1) and harvest index (−62%) vs. Margherita, yet still reaching an overall comparable plant grain yield (Table S8). In WH7, the four genotypes were even more similar for all traits (Tables S8 and S12).

### 3.3. Correlations between Yield and Physiological Traits under Stress

**Figure 7:**
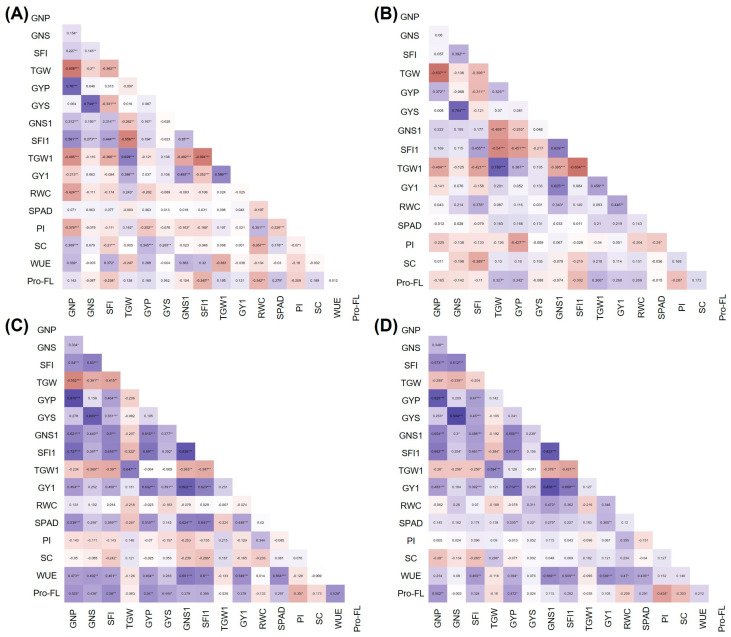
Heatmaps of Pearson’s correlation coefficients (r) between pairs of yield-related traits and physiological parameters under (**A**) control condition, (**B**) 3-day heat stress, (**C**) combined water and 3-day heat stress (WH3); and (**D**) combined water and 7-day heat stress (WH7). Violet and brown colours correspond to positive and negative correlation, respectively. The saturation of colours reflects the absolute value of r. *, ** and *** indicate *p* < 0.05, *p* < 0.01, and *p* < 0.001 significance levels, respectively. For trait acronyms, see the Materials and Methods section.

Pearson’s correlation heatmaps for control, HS, WH3 and WH7 treatments are reported in Figure 7. Under all the conditions considered, yield per spike and per plant (GY1, GYS, GYP) were highly positively correlated with grain number (GNS1, GNS, GNP; r = 0.37 **- 0.90 ***), the latter being in all cases negatively correlated to grain weight (TGW1, TGW). Interestingly, only under the HS treatment (Figure 7B) were GY1 and GYP also positively correlated to TGW1 and TGW (r = 0.39 ** and 0.46 ***, respectively). Among the physiological traits positively correlated to yield under stress conditions, this was the case for RWC, showing a significant correlation with GY1, SFI and GNS1 in the HS treatment (r = 0.45 **, r = 0.38 * and r = 0.34 *, respectively, Figure 7B), confirming the importance of leaf hydration for good productivity. In control conditions only (Figure 7A), stomatal conductance (SC) was correlated with yield (r = 0.35 ***). With the addition of water-deficit to heat stress in WH3 and WH7 treatments (Figure 7C,D), physiological parameters such as SPAD and WUE turned out to be positively correlated to yield at both the main culm and whole plant levels (r = 0.34 **–0.55 ***), revealing their contribution to overcoming heat and drought combination by the tested genotypes. Interestingly, in the most extreme WH7 condition (Figure 7D), SC was shown to be negatively correlated with grain number-related traits GNP (r = −0.28 *) and SFI (r = −0.28 *), which, in turn, were highly positively related to the final yield (r = 0.83 *** and 0.47***, respectively). A positive correlation was also observed between RWC and GNS1 (r = 0.34 *) in WH7 conditions, confirming the importance of RWC in grain number production in harsh environments. Regarding the proline content in flag leaf (Pro-FL), this turned out to be positively correlated to GYP under HS (r = 0.34 *; Figure 7B), as well as WH3 (r = 0.54 **; Figure 7C) and WH7 (r = 0.47 *; Figure 7D) conditions.

### 3.4. Principal Components Analysis (PCA) of Yield-Related Traits under Stress

**Figure 8:**
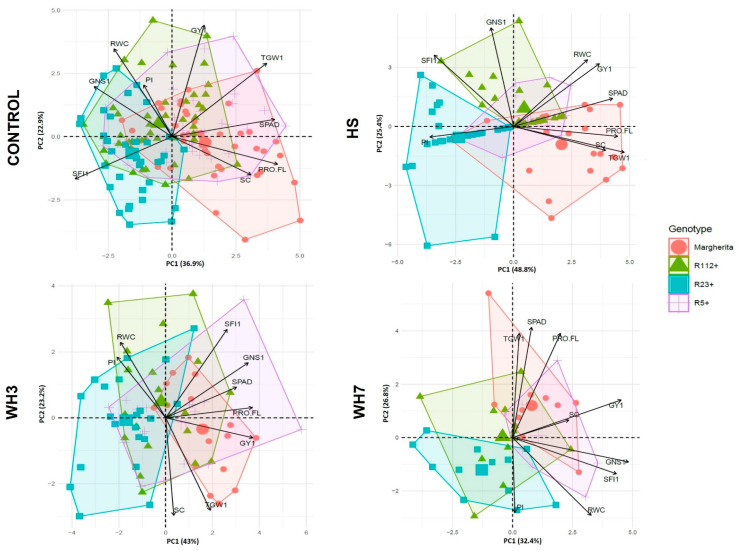
Principal component analysis (PCA) for physiological and yield parameters of the main culms of the three NIRLs+ and cv. Margherita, in control and three stress conditions, i.e., heat stress (HS), water-deficit + 3-day heat stress (WH3) and water-deficit + 7-day heat stress (WH7). The acronyms of the vectors correspond to traits described in the Materials and Methods section.

In order to identify the recombinants with the best productive potential under different stress conditions and traits that mostly affect yield, PCA analyses were performed using a subset of traits of the main culm, the one primarily targeted by stress application at anthesis (Table S13 and Figure 8). The selected traits were those enhanced by the presence of one or more *Th. ponticum* segments as from the ANOVA analyses performed. The first two components of the PCAs explained 59.8%, 74.2%, 66.2% and 59.2% of the total variance for the control, HS, WH3 and WH7 conditions, respectively (Figure 8). Under non-stressed conditions, PC1 (principal component 1) was largely and positively related to GY1, TGW1 and SPAD, and to a lesser extent to Pro-FL and SC. Instead, PC1 was negatively related to GNS1, SFI1, RWC and less to PI. A strong overlap between the genotype clusters could be observed under control conditions, indicating similarities between the alien segment-carriers and the heat tolerant cv. Margherita in the absence of stress. Overall, PCA showed that differences between genotypes in all four conditions were mainly determined by their grain yield (GY1) and 1000-grain weight (TGW1), as these two traits were commonly found on the positive side of PC1 (x axis). Only under water-limited conditions (WH3 and WH7) did PC1 (43% and 32.4% of total variance, respectively) also indicate a positive relation of yield with grain number and spike fertility (GNS1, SFI1), and the positive contribution of the same parameters was confirmed by PC2 (23.2% of total variance) in the WH3 case. In all conditions, physiological traits such as Pro-FL and SC were positively related to GY1, and for SC this relation was particularly clear-cut under the most severe WH7 stress (see the similar angle between the two traits in Figure 8). The importance of leaf hydration (RWC) was highlighted mainly under stress conditions and was closely related to photosynthetic efficiency (PI), as shown by their consistent and close clustering (see PC2 in the control, WH3 and WH7 conditions). Each stress condition was characterized by the specific modulation of relations between parameters. Under HS, PC1 explained 48.8% of the total variance, underlining a strong interrelation between TWG1 and SC, and between GY1 and RWC. In the most extreme WH7 conditions, all physiological and yield-related traits showed a high and positive interrelatedness on PC1, suggesting that the plant productivity under these conditions depends on all the resources available. Based on the observed results under stress, the biplots revealed that, due to TGW1 values, R23+ (light blue cluster, Figure 8) was the most different and low-yielding genotype in all cases, while Margherita (red cluster) was confirmed to be better performing vs. the others, especially under HS only. Under HS alone, due to a higher grain number, R112+ (green cluster) revealed a slightly better yielding ability than the other two recombinants. In contrast, genotype grouping under combined stress showed R5+ (violet cluster) to have an overall comparable (WH7) or better (WH3) performance than Margherita for the main culm traits analysed.

### 3.5. Stress Indices and Tolerance Ranking

The nine stress indices were calculated using the values of GYP in control (Yp) and stress (Ys) conditions (Table 5). The variety Margherita, known for its good performance in hot environments [55], was the best genotype under the present HS conditions for many stress indices, such as MP, GMP, HM, STI and YI (Table 1). After the ranking analysis, Margherita also had the lowest value of the overall average of ranks (AR), which qualifies it as the most tolerant genotype in the HS condition (AR = 2.82), with R5+ (AR = 3.18) and R112+ (AR = 3.73) ranking second and third, respectively. However, according to the TOL, SSI, YSI and RSI indices, reported as most suitable to discriminate well-performing wheat genotypes in heat stressed environments [62], R112+ resulted as the best performing of all genotypes under HS. In the combined WH3 treatment, Margherita was confirmed to be the most tolerant genotype, as indicated by the lowest AR (2.36) and the highest values, as in the HS conditions, of the GMP, HM, STI and YI indices. The second- and the third-ranking genotypes were R5- and R5+ (AR = 2.45 and 3.45, respectively). However, under the most intense WH7 conditions, it was R5+ that resulted as the top ranking line, with high values for the TOL, SSI, YI, YSI and RSI indices and an AR value (1.73) corresponding to less than 60% of the second-ranking Margherita (Table 5). 

## 4. Discussion

In the present work, the yield performance and the physiological response of three DW-*Th. ponticum* NIRLs, having 23%, 28% and 40% of alien 7el_1_L chromatin on their 7AL telomeric region, were evaluated following the application of heat stress (HS) alone and in combination with water-deficit stress (WH) under controlled conditions. Overall, the presence of each of the three alien segments was revealed to be a stabilizing or even enhancing factor for various productivity components, which makes the three NIRLs useful sources for improving wheat abiotic stress tolerance. The results not only largely confirmed previously identified effects on yield-related traits of the same *Th. ponticum* segments when NIRLs were field trialled [23,24,63,64], but also provided novel insights into the stress adaptive mechanisms of the tested recombinants. The present research, based on controlled and targeted stress application at the critical anthesis phase, has for the first time enabled us to minimize interactions with non-controlled factors and, hence, allowed a more precise dissection and appraisal of the stress effects on yield and related physiological parameters. As a result, different response mechanisms could be identified that appeared to be genotype-dependent, and thus ascribable to specific *Th. ponticum* 7el_1_L chromosome segments individually present on the NIRLs’ 7AL arms. To achieve this, the use of near-isogenic lines, carrier (NIRLs+) and non-carrier (NIRLs-) of a given alien segment in the common genetic background of cv. Simeto, was an essential tool. In addition to this, the set of tested physiological parameters allowed the evaluation of key plant features for yield formation, i.e., water status (RWC), photosynthetic potential (SPAD) and efficiency (Fv/Fm, PI), stomatal conductance (SC) and stress sensing and signalling (Pro content). All these mechanisms, greatly affecting plant vitality and fertility, play a fundamental role in tolerance acquisition, being strongly modulated by heat and drought stress [65].

As a whole, under HS, the most important traits affecting yield in association with *Th. ponticum* introgressions were grain weight (GW) and grain number (GN), which exhibited a positive correlation with GY at both the main culm and whole plant levels (Figure 7). In particular, GW of the main culm (TGW1), the one targeted for stress application, turned out to be a major determinant of the observed differences between the genotypes (Figure 8). Even if in wheat it is the seed set that is mostly affected by heat stress at anthesis [66], GW was greatly impactful on yield in our HS experiment, possibly due to the procedure followed in stress application. In fact, a 3-day heat application at anthesis could have reduced GN, with the consequent nutrients allocation being concentrated on the set seeds. On the other hand, GN as a sink trait is the key component determining yield in cereals [67], and in wheat, especially under hot conditions, it was shown to represent the limiting factor in raising yield, with most cultivated genotypes resulting as sink-limited (e.g., [55]). Although GN is generally determined in pre-anthesis phases, it can be negatively affected by abiotic stresses at anthesis, in particular during anther dehiscence [67]. Failure to release pollen from anthers, but also reduced pollen viability, limit the successful fertilization and final seed formation [66,67,68]. *Th. ponticum* 7el_1_L introgressions in both bread and durum wheat were shown to contribute to the increase in GN in heat-affected natural environments [23,28,64,69], and those tested here have been validated as a source of useful grain traits for yield stability under such stress. 

Under the combined WH stress, most decisive and discriminating yield traits turned out to be GN and the positively correlated spike fertility index (SFI, Figure 7). Moreover, as highlighted by PCA (Figure 8), all physiological parameters displayed an essential role in yield preservation, emphasizing how strongly the maintenance of flag leaf physiology impacts on wheat production under adverse conditions (see § 3.2). Normally, the combination of heat and drought stress has a negative and cumulative impact on plant phenology and physiology [37,70]. However, the impact on yield components depends on both stress duration and severity and on the plant developmental stage. In wheat, episodes of drought and heat stress around anthesis, as in our experimental design, generally reduce the final seed set in a more severe way than the heat stress alone does [37]. Moreover, if water limitation is prolonged during grain filling, it reduces photosynthesis, induces early senescence, shortens the grain-filling period and can concomitantly increase the remobilization of assimilates from leaves and stems to the grains, raising the grain filling rate [71]. In the WH experiments performed here, a strong impact on GNP was detected (from −20 to −70% in WH7 vs. control conditions, Figure 6A and Tables S11 and S12), which was remarkably superior to that detected under the single HS (Tables S3 and S4). In contrast, an overall increase in TGW was observed in all three experiments, but its slight increment detected in WH3 and WH7 was not able to compensate for the decrease of all other yield components. Regarding the physiological parameters, those associated with plant water management (WU, WUE, SC) were especially critical to achieve tolerance and preserve yield (see also [37,42]).

Considering the stress effects more specifically concerning each recombinant line, the 23%-long segment present in R5+ was found to be associated with the highest yield stability and overall physiological activity across all stress conditions examined here. The alien segment favoured R5+ particularly under the combined stress experiments (Figure 8), when considerable Pro accumulation in the flag leaf (Pro-FL) and an efficient stress perception leading to stomatal closure were observed (Figure 5, Tables S7, S8, S11 and S12). The Pro increase was higher than that of the heat tolerant cv. Margherita and was strongly and positively correlated with GYP in all stress experiments performed here (Figure 7) and in other studies (e.g., [72]). R5+ proved to be the top performing genotype, especially in the most extreme WH7 condition, when it showed the highest values for WUE, PI and HI (Figure 6), besides that of GW and final GY (Tables S9–S12). The enhanced TGW of R5+ was previously observed under rainfed field conditions [24,73], where drought and heat naturally occurred at the time of grain filling. The R5+ advantage for HI confirms previous observations in a low-yielding environment of South Australia, characterized by a short season, low rainfall, high temperatures and recurrent heat waves [23]. This suggests a putatively higher ability of R5+ to allocate dry matter more towards grains than biomass under unfavourable conditions. It is well established that, in water-limited environments, plants put in place water-saving mechanisms, including regulation of stomatal aperture and consequent reduction in the rate of water vapour dispersion from leaves [46,47,48]. The same tolerance mechanism was also observed under a combination of heat and water-deficit conditions, when plants generally close stomata to limit water loss [74]. In our observations, the combination of prompt stomata closure together with an efficient use of available water for grain production (WUE) have likely given R5+ a remarkable yield advantage (Figure 5B and Figure 6A, Tables S11 and S12). The general high yield stability exhibited by R5+ in the present HS and WH experiments is in agreement with a previous observation of the highest yield gains (up to 30%) that R5+ displayed vs. R112+ and R23+ across a range of nine contrasting and mostly stressful environments [23]. 

Regarding the R112+ recombinant line, a positive effect of its 28%-long *Th. ponticum* segment was evident under HS, with all yield-related traits revealing their high stability (Table S3). Compared with Margherita and the other two NIRLs, R112+ largely compensated for its lower TGW with superior values for all parameters describing grain number (GNS, GNSL, SFI, GNP). The superiority of R112+ for GN was previously observed in multi-environment rainfed trials, which indicated R112+ to be best suited to environments characterised by heat stress not accompanied by major water scarcity [23,63,64]. In the HS experiment, R112+, as R5+, maintained all yield parameters unchanged in the stressed vs. control conditions (Table S3). Both recombinant NIRLs, like the heat tolerant cv. Margherita (Figure 4B), displayed higher RWC under HS as compared to their NIRL- controls (Figure 4A). In addition, the *Th. ponticum* chromatin in common between R112+ and R23+ (Figure 1) was associated with a high photosynthetic efficiency under HS, as shown by their PI index (Figure 4B). PI remained unchanged (R112+) or even increased (R23+) in HS vs. control conditions, suggesting the remarkable efficiency of the photosynthetic apparatus when coping with the HS condition. A high photosynthetic potential and rate of R112+ were previously observed in field trials across several environments, in which enhanced values for traits such as flag leaf area, chlorophyll content [63,64] and photosynthetic efficiency [24] were detected. On the other hand, while R112+ was one of the best performing genotypes under the HS alone, the same was not observed under the combined WH stress. This could already be perceived in the WH3 treatment (Tables S5–S8) and became more evident with prolonged stress duration (WH7), particularly in comparison with the behaviour of R5+ (Tables S9–S12). The unchanged amount of water use (WU) and decreased WUE in WH7 vs. the control condition (Figure 6A, Tables S9–S12) are suggestive of the higher amounts of water required by R112+ vs. the other genotypes in both optimal and stress conditions, and of lower efficiency in activating water-saving mechanisms that are fundamental to cope with water-limited conditions [75].

The NIRL carrier of the longest *Th. ponticum* chromosome segment, R23+, resulted as the most sensitive among the three recombinants under both HS and combined WH stress, with many of the yield traits significantly reduced with respect to non-stress conditions (Tables S3, S7 and S11). This is at least in part ascribable to linkage drag associated with the most proximal portion of its alien segment, not shared with the other NIRLs+ [23,63,64], as well as to its genetic background, in common with R23–, but somewhat less isogenic with respect to the remaining NIRLs [23]. Nonetheless, apart from the low absolute values, when compared to its R23- sibling, R23+ confirmed some exceptionally positive effects of its 7el_1_L segment on spike fertility (SFI) and plant GN. SFI, in particular, remained significantly higher in R23+ vs. R23- under both control (≈ +45%) and, notably, stress conditions (+105% to +230%, depending on the stress treatment, Tables S3, S7 and S11). This result is in line with the high increase of GN that R23+ displayed in dry environments (South Australia, Morocco), characterized by both heat and drought stress at critical developmental phases [23]. Higher GN, GNSL and SFI, consistently displayed by field grown R23+ plants, were tentatively associated with the recombinant’s constitutively higher floret survival rate [23,63,64]. This hypothesis is strongly sustained by the present report, and further studies are underway to determine the genetic and physiological bases of R23+ reproductive resilience. SFI has been proposed as a promising trait to select for the best genotypes in wheat breeding programs [76], as increased SFI implies improved partitioning of assimilates, fundamental to support wheat yield potential in hot environments [77]. Besides SFI, HI and GNP also turned out to be statistically superior in R23+ vs. R23- in the WH7 experiment, suggesting that the presence of the 40%-long 7el_1_L chromosome segment might cause preferential nutrients allocation in seeds rather than in spike chaff under unfavourable conditions. Moreover, lower water use by R23+ vs. R23-, a behaviour that may have contributed to the maintenance of high spike fertility in the former, was for the first time revealed under WH7 stress (Figure 6A). A further new insight concerned Pro accumulation in R23+ spike tissues (Pro-SP). This NIRL+ was the genotype with the highest Pro-SP in absolute terms under both control and HS conditions and, unlike its NIRL- sib, maintained Pro-SP unchanged after the HS treatment (Figure 4A). Whether Pro accumulation in spikes is a specific R23+ mechanism to cope with stress and might be correlated with its increased fertility under HS remains to be further elucidated.

Our analysis of the Pro content in both leaves and spikes of the durum wheat-*Th. ponticum* recombinant lines, under control and stress conditions, showed the dynamics of Pro accumulation in spikes to be more complex. In fact, in line with many other studies on leaves of heat- and/or drought-stressed wheat plants (e.g., [52,72]), Pro-FL was very similar across the genotypes under control conditions, and exhibited an overall increase under all applied stresses, with major increments detected in R5+ and Margherita (Tables S3, S4, S7, S8, S11 and S12). As for Pro-SP, the present experiments clearly showed that in all conditions much larger Pro amounts were present in spikes than in leaves (10–30-fold greater across genotypes), confirming previous evidence in wheat and barley [78,79]. However, while showing only minor variations under HS alone (Figure 4), when the three days of heat stress were accompanied by a water-deficit condition (WH3 experiment), a considerable decrement of Pro-SP was detected in all genotypes (Figure 5B). The rate and direction of Pro accumulation under combined heat and drought stress are still unclear and are probably connected with the intensity/timing of stress application, as well as with the plant tissue analysed. For example, Arabidopsis plants subjected to a combination of heat and drought accumulate sucrose instead of proline, suggesting that sucrose replaces proline as the major osmoprotectant, probably to safeguard the overactive and sensitive mitochondria from the potentially toxic pyrroline-5-carboxylate, an intermediate in Pro biosynthesis and degradation [80]. Other studies on various species provide controversial results for Pro accumulation under individual or combined heat and drought stresses (e.g., [81,82]). Proline’s role in stressed spike tissues is poorly understood, not least because most analyses have been focused on leaves. A recent work on the response to drought stress by different wheat organs [78] underlined how, compared with flag leaf, smaller amount of ROS and higher amounts of antioxidant enzymes and proline were accumulated in spike organs under stress, associating these response mechanisms with a better water status, higher photosynthesis and lower membrane damage. As above recalled, also in our experiments both control and stressed spikes exhibited a superior Pro content than in flag leaves in absolute terms, even if the combination of heat and drought caused a general Pro decrease in spikes compared to unstressed conditions. Given the relevance of spike traits for yield performance under stress, and the special stress-responsive behaviour apparently associated with this organ (e.g., [83]), the mechanisms exerted at the spike level by our recombinant lines will be further investigated in a future study.

## 5. Conclusions

As a whole, the results of the present study showed that, among the three chromosomally engineered durum wheat-*Th. ponticum* recombinant lines, the one possessing the smallest amount of alien 7el_1_L introgression, namely R5+, already known to possess very effective disease resistance genes within its alien segment [19,54], exhibited excelling yield stability across all the applied abiotic stress conditions. This was primarily due to highly efficient plant water use, but also to an effective flag leaf physiology (chlorophyll activity, Pro accumulation) and high grain weight. R5+ also proved to be comparable for productivity and some physiological adaptive mechanisms to the elite heat tolerant line Margherita, which validates its readiness to be introduced in breeding programs for abiotic-stress prone environments, such as those of the Mediterranean basin. Further confirmation of the very promising behaviour of R5+, besides that of R5-derived secondary recombinants, and, to some extent, of R112+, comes from the recent results of field trials carried out in the natural stressful conditions of Algeria, Italy and Turkey [84,85]. No doubt, improving abiotic stress tolerance in wheat is essential to achieve yield stability and to decrease the yield gap under changing climate [1,6,31,86]. The evidence reported here substantiates how, for this and other key targets, the use of chromosome engineering to harness wild relatives’ useful attributes [19,20,21,22] is an effective strategy to develop stable and functional genotypes for large-scale applications.

## Figures and Tables

**Table 1:** Stress indices calculated with the iPASTIC software with corresponding formulas [60].

Index	Formula
Tolerance index (TOL)	TOL = Yp − Ys
Mean productivity (MP)	MP = (Yp + Ys)/2
Geometric mean productivity (GMP)	GMP = √(Ys × Yp)
Harmonic mean (HM)	HM= 2 (Ys × Yp)/(Ys + Yp)
Stress susceptibility index (SSI)	SSI = 1 − (Ys/Yp)/1 − (Ȳs/Ȳp)
Stress tolerance index (STI)	STI = (Ys × Yp)/(Ȳp)²
Yield index (YI)	YI = Ys/Ȳs
Yield stability index (YSI)	YSI = Ys/Yp
Relative stress index (RSI)	RSI = (Ys/Yp)/(Ȳs/Ȳp)

**Table 2:** ANOVA mean squares for physiological, biochemical and yield-related traits measured under control and HS treatment: (A) comparison between alien segment carrier (NIRLs+) and non-carrier (NIRLs-) lines, and (B) comparison between NIRLs+ and cv. Margherita. *, **, and *** indicate significance at *p* < 0.05, *p* < 0.01 and *p* < 0.001 levels, respectively (df, degrees of freedom; G, genotype; T, treatment). For trait acronyms, see the Materials and Methods section.

	A				B			
Factors	G	T	G × T	Residuals	G	T	G × T	Residuals
df	5	1	5		3	1	3	
SPAD	718.3 ***	149.9 ***	52.1 ***	11.2	750.6 ***	119.3 **	7.3	13.4
SC	24341.2 ***	280889.3 ***	14015.7 **	3489.9	105054.2 ***	491996.4 ***	38365.4 ***	3525.7
TL	1.12	2.15	2.14	1.02	2.40 *	9.71 ***	9.58 ***	0.76
Fv/Fm	0.00008	0.00028 *	0.00005	0.00006	0.00004	0.00004	0.00010	0.00006
Fv/F0	0.15	0.29 *	0.07	0.07	0.07	0.06	0.09	0.06
PI	15.2 ***	1.41	7.50 *	2.38	9.47 **	1.03	8.11 **	1.87
RWC	0.001 ***	0.0001	0.0005 ***	0.00005	0.001 ***	0.0003 *	0.0005 ***	0.0001
Pro-FL	9.47	145.1 ***	5.65	4.61	34.9 **	196.4 ***	6.80	5.62
Pro-SP	2737.6 ***	1468.1 *	803.9 *	293.4	6369.7 ***	149.3	631.0	380.4
GNS1	60.7	25.4	158.2 **	36.5	162.9 **	252.8 **	169.50**	31.2
GNSL1	0.73 ***	0.10	0.50 *	0.17	0.39	0.73 *	0.58 *	0.16
SFI1	1884.6 ***	291.6	555.5 *	181.9	3554.9 ***	1368.4 **	1165.25***	181.7
TGW1	385.9 ***	85.6	91.9	47.5	843.3 ***	166.6	257.1 **	46.1
GYS1	0.34 *	0.04	0.27 *	0.12	0.45 *	0.12	0.16	0.11
GNS	171.4 *	383.6 *	123.1	65.1	183.4 *	794.5 ***	195.4 *	60.9
GNSL	1.58 ***	2.07 **	0.49	0.25	1.03 **	4.68 ***	0.77 *	0.24
SFI	5718.6 ***	3357.2 **	1506.1 ***	316.2	20441.3 ***	6067.4 ***	2997.9 ***	365.5
GYS	0.49 *	0.001	0.34	0.18	1.57 ***	0.20	0.38	0.17
TNP	1.87 *	6.23 **	1.60 *	0.64	0.81	3.38 *	1.30	0.49
PTP	2.94 ***	1.35	0.33	0.49	3.23 ***	0.85	0.32	0.50
GNP	2809.1 ***	8015.9 ***	1224.1 **	375.7	868.9	8335.6 ***	2391.6 ***	359.3
TGW	381.6 ***	282.1 *	103.9	46.6	854.2 ***	301.1 *	257.8 **	48.7
GYP	1.74 ***	4.23 ***	1.59 ***	0.36	9.07 ***	7.03 ***	2.08 ***	0.32
H	2191.3 ***	401.9 ***	49.8	26.3	1110.1 ***	171.2 **	39.1	17.7

**Table 3:** ANOVA mean squares for physiological, biochemical and yield-related traits measured under control and combined water-deficit and 3-day HS (WH3): (A) comparison between alien segment carrier (+) and non-carrier (−) NIRLs, and (B) comparison between NIRLs+ and cv. Margherita. *, **, and *** indicate significance at *p* < 0.05, *p* < 0.01 and *p* < 0.001 levels, respectively. For trait acronyms, see the Materials and Methods section.

	A				B			
Factors	G	T	G × T	Residuals	G	T	G × T	Residuals
df	5	1	5		3	1	3	
SPAD	1017.7 ***	168.9	126.9 *	55.6	671.1 ***	408.5 **	139.1	58.2
SC	10722.8	154764.8 ***	16595.3 *	5440.0	53776.6 ***	359666.3 ***	32983.9 ***	4722.6
TL	4.52	70.80 ***	1.64	2.17	6.09 *	71.12 ***	0.64	1.66
Fv/Fm	0.00017 ***	0.00001	0.00005	0.00004	0.0002 ***	0.0000001	0.0001 *	0.00004
Fv/F0	0.25 ***	0.003	0.15 *	0.05	0.34 ***	0.08	0.09	0.05
PI	11.89**	3.24	2.58	3.51	23.8 ***	23.8 **	10.7 *	3.1
RWC	0.00002	0.00015	0.0001	0.00009	0.00003	0.00006	0.0001	0.0001
WU	137.4	8958.2 ***	439.8 ***	58.6	1049.5 ***	11465.6 ***	670.9 ***	61.4
WUE	0.0005 **	0.0003	0.001 ***	0.0002	0.0001	0.0001	0.0007 *	0.0002
Pro-FL	1.15	2.12	2.13 *	0.62	2.25 *	6.08 **	2.16 *	0.59
Pro-SP	701.5 *	33878.8 ***	475.6	243.0	1132.9 *	15349.5 ***	996.3 *	271.1
GNS1	375.9 ***	4700.6 ***	193.6 ***	39.0	69.5	2072.7 ***	268.6 ***	32.6
GNSL1	2.22 ***	19.1 ***	1.00 **	0.28	0.52	6.83 ***	1.23 **	0.27
SFI1	2616.9 ***	12260.6 ***	1778.0 ***	268.3	159.9	5843.2 ***	1596.3 ***	209.5
TGW1	482.7 ***	803.3 ***	80.6	53.1	455.7 ***	32.1	8.73	54.8
GYS1	0.90 ***	6.17 ***	0.40 **	0.12	0.82 ***	3.21 ***	0.21	0.11
GNS	364.5 ***	6432.5 ***	216.9 **	57.9	47.9	2614.0 ***	252.2 **	57.8
GNSL	1.94 ***	25.1 ***	0.84 *	0.31	0.24	9.80 ***	0.78	0.32
SFI	4931.4 ***	14469.5 ***	1991.1 ***	281.6	1746.5 ***	5137.5 ***	1217.9 **	293.6
GYS	0.61 **	9.00 ***	0.31	0.16	0.71 **	3.74 ***	0.13	0.15
TNP	3.40 ***	0.31	4.22 ***	0.50	6.25***	5.11 **	3.93 **	0.67
PTP	3.45 ***	2.22 *	2.84 ***	0.48	6.13 ***	6.08 **	2.52 **	0.60
GNP	4203.4 ***	22969.3 ***	5385.4 ***	204.2	5182.7 ***	24323.4 ***	6268.4 ***	268.2
TGW	357.3 ***	734.1 ***	22.6	30.9	298.4 ***	294.2 **	17.1	32.9
BM	45.8 ***	114.5 ***	16.3 **	3.97	61.7 ***	173.5 ***	13.3 **	3.69
HI	0.06 ***	0.05 ***	0.03 ***	0.004	0.04 ***	0.07 ***	0.02 **	0.003
GYP	4.54 ***	34.2 ***	8.44 ***	0.71	7.03 ***	41.3 ***	7.9 ***	1.02
H	1002.7 ***	52.4	22.9	16.4	765.9 ***	33.5	55.1 *	16.1

**Table 4:** ANOVA mean squares for physiological, biochemical and yield-related traits measured under control and combined water-deficit and 7-day HS stress (WH7): (A) comparison between alien segment carrier (+) and non-carrier (−) NIRLs, and (B) comparison between NIRLs+ and cv. Margherita. *, **, and *** indicate significance at *p* < 0.05, *p* < 0.01 and *p* < 0.001 levels, respectively. For trait acronyms, see the Materials and Methods section.

	A				B			
Factors	G	T	G × T	Residuals	G	T	G × T	Residuals
df	5	1	5		3	1	3	
SPAD	515.6 ***	583.5 ***	84.9 *	28.1	265.2 ***	602.4 ***	48.7	36.5
SC	15069.4 **	760720.9 ***	9298.6 *	3700.3	34393.1 ***	614880.3 ***	10975.7	4858.0
TL	2.02	73.7 ***	7.08 *	2.94	10.0 **	52.4 ***	3.12	2.46
Fv/Fm	0.00004	0.0005 **	0.00005	0.00006	0.00007	0.0003 *	0.00005	0.00006
Fv/F0	0.09	0.68 **	0.06	0.07	0.13	0.47 *	0.05	0.07
PI	4.64	46.50 ***	3.05	3.37	3.94	18.5 *	17.9 **	3.80
RWC	0.0004 *	0.00004	0.0002	0.0001	0.0002	0.001 **	0.0002	0.0001
WU	205.2 **	5666.1 ***	419.7 ***	54.0	1033.9 ***	6972.3 ***	678.1 ***	33.4
WUE	0.0004 **	0.002 ***	0.001 ***	0.0001	0.0001	0.003 ***	0.0007 ***	0.0001
Pro-FL	3.28 *	23.8 ***	2.10	1.01	5.17 *	37.3 ***	1.18	1.40
GNS1	335.8 ***	6199.9 ***	209.9 ***	39.4	12.8	4117.6 ***	225.2 **	43.6
GNSL1	2.27 ***	25.6 ***	1.00 ***	0.20	0.30	17.7 ***	0.80 **	0.25
SFI1	2620.6 ***	14652.8 ***	1660.3 ***	220.6	313.2	6230. 6 ***	2093.9 ***	235.9
TGW1	489.9 ***	28.4	16.4	37.9	590.0 ***	0.34	2.60	42.7
GYS1	0.82 ***	12.7 ***	0.40 **	0.09	0.87 ***	8.61 ***	0.23	0.09
GNS	221.7 **	9992.5 ***	190.3 *	64.1	42.3	9909.4 ***	209.3 *	65.3
GNSL	1.64 ***	40.3 ***	0.72 *	0.29	0.13	41.3 ***	0.73	0.33
SFI	3232.2 ***	28549.0 ***	2528.4 ***	276.6	1141.1 *	19765.9 ***	3232.8 ***	331.7
GYS	0.78 ***	18.6 ***	0.25	0.14	0.76 **	17.7 ***	0.17	0.14
TNP	5.17 ***	1.39	3.35 ***	0.46	5.78 ***	3.08 *	5.08 ***	0.60
PTP	4.72 ***	3.43 **	2.55 ***	0.42	4.83 ***	4.23 **	3.82 ***	0.53
GNP	5414.3 ***	35354.0 ***	4694.8 ***	200.9	6196.3 ***	36513.4 ***	6394.2 ***	282.2
TGW	412.3 ***	194.3 **	5.6	18.8	515.4 ***	155.3 **	3.83	17.5
BM	20.7 ***	177.2 ***	32.6 ***	3.5	19.2 **	279.3 ***	37.3 ***	3.76
HI	0.03 ***	0.07 ***	0.01 ***	0.002	0.01 **	0.05 ***	0.01	0.003
GYP	3.57 ***	69.5 ***	9.6 ***	0.58	4.13 **	78.0 ***	12.6 ***	0.85
H	860.7 ***	251.0 ***	59.3 *	19.6	741.4 ***	495.3 ***	49.1 *	16.6

**Table 5:** Stress indices’ values and ranking of the genotypes tested in the three stress experiments performed: HS, heat stress; WH3, water-deficit + 3-day heat stress; and WH7, water-deficit + 7-day heat stress. The best value for each index appears in bold. For acronyms of the stress indices see the Materials and Methods section; SR, sum of ranks; SD, standard deviation; AR, average of ranks.

Treatment	Genotype	Stress Tolerance Indices																								
		Yp		Ys		TOL		MP		GMP		HM		SSI		STI		YI		YSI		RSI		SR	SD	AR
		Value	Rank	Value	Rank	Value	Rank	Value	Rank	Value	Rank	Value	Rank	Value	Rank	Value	Rank	Value	Rank	Value	Rank	Value	Rank			
HS	Margherita	5.65	1	4.94	1	0.71	6	5.30	1	5.29	1	5.27	1	1.38	6	1.48	1	1.25	1	0.87	6	0.96	6	31	2.52	**2.82**
	R5+	4.72	3	4.20	2	0.52	5	4.46	2	4.45	2	4.45	2	1.22	5	1.05	2	1.06	2	0.89	5	0.98	5	35	1.47	**3.18**
	R5-	3.75	6	3.76	4	−0.01	2	3.76	5	3.75	5	3.75	5	−0.04	2	0.75	5	0.95	4	1.00	2	1.10	2	42	1.54	3.82
	R112+	3.67	7	3.84	3	−0.17	1	3.75	6	3.75	6	3.75	6	−0.52	1	0.74	6	0.97	3	1.05	1	1.15	1	41	2.49	**3.73**
	R112-	4.06	4	3.75	5	0.31	4	3.90	4	3.90	4	3.90	4	0.84	4	0.80	4	0.95	5	0.92	4	1.02	4	46	0.40	4.18
	R23+	4.73	2	3.51	7	1.22	7	4.12	3	4.08	3	4.03	3	2.84	7	0.88	3	0.89	7	0.74	7	0.82	7	56	2.21	5.09
	R23-	3.84	5	3.66	6	0.19	3	3.75	7	3.75	7	3.75	7	0.53	3	0.74	7	0.93	6	0.95	3	1.05	3	57	1.83	5.18
WH3	Margherita	4.39	2	3.06	1	1.33	5	3.73	2	3.67	1	3.61	1	0.85	4	1.10	1	1.36	1	0.70	4	1.08	4	26	1.57	**2.36**
	R5+	2.95	4	2.49	3	0.47	3	2.72	4	2.71	4	2.70	4	0.44	3	0.60	4	1.10	3	0.84	3	1.31	3	38	0.52	**3.45**
	R5-	2.60	7	2.98	2	−0.38	1	2.79	3	2.78	3	2.78	3	−0.42	1	0.63	3	1.33	2	1.15	1	1.78	1	27	1.75	**2.45**
	R112+	2.94	5	1.87	5	1.07	4	2.40	6	2.34	6	2.28	6	1.03	5	0.45	6	0.83	5	0.63	5	0.99	5	58	0.65	5.27
	R112-	2.80	6	2.46	4	0.34	2	2.63	5	2.63	5	2.62	5	0.34	2	0.56	5	1.09	4	0.88	2	1.37	2	42	1.54	3.82
	R23+	5.62	1	1.86	6	3.76	7	3.74	1	3.23	2	2.79	2	1.88	6	0.85	2	0.82	6	0.33	6	0.51	6	45	2.43	4.09
	R23-	3.17	3	1.04	7	2.13	6	2.10	7	1.81	7	1.56	7	1.89	7	0.27	7	0.46	7	0.33	7	0.51	7	72	1.21	6.55
WH7	Margherita	4.39	2	2.12	2	2.27	6	3.25	2	3.05	1	2.86	1	1.05	5	0.76	1	1.19	2	0.48	5	0.95	5	32	1.92	**2.91**
	R5+	2.95	4	2.37	1	0.59	1	2.66	3	2.64	2	2.63	2	0.40	1	0.57	2	1.33	1	0.80	1	1.58	1	19	1.01	**1.73**
	R5-	2.60	7	1.92	4	0.68	2	2.26	6	2.23	6	2.21	4	0.53	2	0.41	6	1.08	4	0.74	2	1.46	2	45	1.92	4.09
	R112+	2.94	5	1.75	5	1.19	4	2.34	5	2.27	5	2.19	5	0.82	4	0.42	5	0.99	5	0.59	4	1.17	4	51	0.50	4.64
	R112-	2.80	6	2.04	3	0.76	3	2.42	4	2.39	4	2.36	3	0.55	3	0.47	4	1.15	3	0.73	3	1.43	3	39	0.93	**3.55**
	R23+	5.62	1	1.16	6	4.45	7	3.39	1	2.56	3	1.93	6	1.61	7	0.54	3	0.66	6	0.21	7	0.41	7	54	2.43	4.91
	R23-	3.17	3	1.06	7	2.11	5	2.12	7	1.83	7	1.59	7	1.35	6	0.28	7	0.60	7	0.34	6	0.66	6	68	1.25	6.18

## Data Availability

The data presented in this study are available in supplementary material here.

## Supplementary Materials

The following supporting information can be downloaded at: https://www.mdpi.com/article/10.3390/plants12040704/s1. Table S1: Heat stress experiment: statistical comparison for G effect performed between the NIRLs (“+” vs. “−”). Table S2: Heat stress experiment: statistical comparison for G effect performed between the NIRLs+ and cv. Margherita. Table S3: Heat stress experiment: statistical comparison for genotype x treatment (G × T) effect performed between the NIRLs (“+” vs. “−”). Table S4: Heat stress experiment: statistical comparison for G × T effect performed between the NIRLs+ and cv. Margherita. Table S5: Combined water-deficit + heat stress experiment, with 3 days of heat stress (WH3): statistical comparison for G effect performed between the NIRLs (“+” vs. “−”). Table S6: Combined water-deficit + heat stress experiment, with 3 days of heat stress (WH3): statistical comparison for G effect performed between the NIRLs+ and cv. Margherita. Table S7: Combined water-deficit + heat stress experiment, with 3 days of heat stress (WH3): statistical comparison for genotype x treatment (G × T) effect performed between the NIRLs (“+” vs. “−”). Table S8: Combined water-deficit + heat stress experiment, with 3 days of heat stress (WH3): statistical comparison for G × T effect performed between the NIRLs+ and cv. Margherita. Table S9: Combined water-deficit + heat stress experiment, with 7 days of heat stress (WH7): statistical comparison for G effect performed between the NIRLs (“+” vs. “−”). Table S10: Combined water-deficit + heat stress experiment, with 7 days of heat stress (WH7): statistical comparison for G effect performed between the NIRLs+ and cv. Margherita. Table S11: Combined water-deficit + heat stress experiment, with 7 days of heat stress (WH7): statistical comparison for genotype x treatment (G × T) effect performed between the NIRLs (“+” vs. “−”). Table S12: Combined water-deficit + heat stress experiment, with 7 days of heat stress (WH7): statistical comparison for G × T effect performed between the NIRLs+ and cv. Margherita. Table S13: Correlations between five principal components’ variants and main culm physiological/yield-related traits. (A) Control condition; (B) Heat stress treatment (HS); (C) Water-deficit + heat stress for 3 days (WH3); (D) Water-deficit + heat stress for 7 days (WH7).
